# Investigation of ternary Zn–Co–Fe layered double hydroxide as a multifunctional 2D layered adsorbent for moxifloxacin and antifungal disinfection

**DOI:** 10.1038/s41598-023-48382-0

**Published:** 2024-01-08

**Authors:** Rehab Mahmoud, Nada M. Kotb, Yasser GadelHak, Fatma I. Abo El-Ela, Ayman Z. Shehata, Sarah I. Othman, Ahmed A. Allam, Hassan Ahmed Rudayni, Amal Zaher

**Affiliations:** 1 Chemistry Department, Faculty of Science, Beni-Suef University, Beni-Suef, 62511 Egypt; 2 Hydrogeology and Environment Department, Faculty of Earth Sciences, Beni-Suef University, Beni-Suef, Egypt; 3 Department of Materials Science and Nanotechnology, Faculty of Postgraduate Studies for Advanced Sciences, Beni-Suef University, Beni-Suef, 62511 Egypt; 4 Department of Pharmacology, Faculty of Veterinary Medicine, Beni-Suef University, Beni-Suef, 62511 Egypt; 5 Department of Food Safety and Technology, Faculty of Veterinary Medicine, Beni-Suef University, Beni-Suef, Egypt; 6 Department of Biology, College of Science, Princess Nourah Bint Abdulrahman University, P.O. BOX 84428, 11671 Riyadh, Saudi Arabia; 7 Department of Biology, College of Science, Imam Muhammad Ibn Saud Islamic University, 11623 Riyadh, Saudi Arabia; 8 Department of Zoology, Faculty of Science, Beni-Suef University, Beni-Suef, 62511 Egypt; 9 Environmental Science and Industrial Development Department, Faculty of Postgraduate Studies for Advanced Sciences, Beni-Suef University, Beni-Suef, Egypt

**Keywords:** Two-dimensional materials, Antifungal agents, Pollution remediation

## Abstract

Layered double hydroxides have recently gained wide interest as promising multifunctional nanomaterials. In this work, a multifunctional ternary Zn–Co–Fe LDH was prepared and characterized using XRD, FTIR, BET, TEM, SEM, and EDX. This LDH showed a typical XRD pattern with a crystallite size of 3.52 nm and a BET surface area of 155.9 m^2^/g. This LDH was investigated, for the first time, as an adsorbent for moxifloxacin, a common fluoroquinolones antibiotic, showing a maximum removal efficiency and equilibrium time of 217.81 mg/g and 60 min, respectively. Its antifungal activity, for the first time, was investigated against *Penicillium notatum, Aspergillus flavus, Aspergillus fumigatus, Aspergillus niger,* and *Mucor fungi* at various concentrations (1000–1.95 µg/mL). This LDH was found to be effective against a variety of fungal strains, particularly *Penicillium* and *Mucor* species and showed zones of inhibition of 19.3 and 21.6 mm for *Penicillium* and *Mucor*, respectively, with an inhibition of 85% for *Penicillium* species and 68.3% for *Mucor*mycosis. The highest antifungal efficacy results were obtained at very low MIC concentrations (33.3 and 62 µg/ml) against *Penicillium* and *Mucor*, respectively. The results of this study suggest a promising multifunctional potential of this LDH for water and wastewater treatment and disinfection applications.

There is an urgent need to develop cost-effective high-performance multifunctional nanomaterials that can be applied in different fields of research such as environmental and/or biomedical applications. These multifunctional nanomaterials can be defined as those able to achieve more than one function or a combined effect through their multiple functionalization or combination with other materials^1^. Nanomaterials have been investigated for numerous environmental and biomedical applications due to their unique properties especially their large surface-to-volume ratio^2,3^. Nanomaterials show wide varieties in terms of their chemistry, structure, morphology, size, surface charge, and many other criteria. Investigating and optimizing the chemistry and structure of nanomaterials can lead to enhanced performance for a given application compared to bulk conventional materials^4^. Multifunctional nanomaterials can even provide better performance due to their added functionality and possible utilization in numerous applications simultaneously. Several types of nanomaterials could be considered as promising multifunctional candidates such as silver nanoparticles, chitosan nanoparticles, graphene and its oxide, metal oxides, and metal organic frameworks, to name a few^4–7^.

One of the families of multifunctional nanomaterials that has gained wide interest recently is layered double hydroxides (LDH)^8^. LDHs are defined as 2D anionic clay materials, similar in structure to brucite, which possess the chemical formula [M(II)(1 − x)M(III)x(OH)2]^x+^(A^n^ −)_x/n_.yH_2_O. M(II) is a divalent cation such as magnesium, copper, or cobalt, while M(III) is a trivalent cation such as aluminium, chromium, or iron, and A^n−^ represents the negative anions such as carbonate, chloride, or nitrate^9^. LDH has attracted much interest as multifunctional adsorbents due to their adequate specific surface area, low toxicity, high adsorption capacity, cheap synthesis, and stable properties^10^. Our research group has reported numerous previous contributions to the field of exploring multifunctional potential of several LDH types that can be used for waste treatment, water disinfection, and biomedical applications. For instance, Mahmoud et al.^11^ prepared ZnFe binary LDH using a simple co-precipitation method and studied its use for methyl orange, methylene blue, and malachite green removal. This adsorbent showed a maximum adsorption capacity of 230.68, 133.29, and 57.34 mg/g for methyl orange, methylene blue, and malachite green, respectively. Furthermore, cobalt, and magnesium were incorporated in the binary ZnFe LDH to produce ternary ZnCoFe and ZnMgFe LDH respectively as reported by Abdel Aziz et al.^12^. These ternary LDH samples were investigated for the disinfection of real dairy wastewater effluents. The antimicrobial activity of ZnCoFe LDH was increased significantly at a concentration of 0.005 mg/L followed by MgZnFe LDH. ZnCoFe was also used as an adsorbent of Ceftriaxone and the spent adsorbent was recycled as an adsorbent for malachite green removal^13^. ZnCoFe was also used for oxytetracycline adsorption showing a maximum adsorption capacity of 240.29 mg/g while the spend adsorbent was recycled for methanol electro-oxidation^14^. Sayed et al.^15^ prepared nickel incorporated ZnCoFe LDH and tested it as an adsorbent for methyl orange removal. This LDH showed an adsorption capacity reaching a maximum of 418 mg/g and an equilibrium time of at 60 min. In addition, this LDH showed a promising antibacterial effect towards both Gram-negative and Gram-positive bacteria. Even the spent adsorbent was reutilized as a promising methanol electro‑oxidation catalyst^16^. In this work, we continue our work and explore the utilization of ZnCoFe LDH as an adsorbent for moxifloxacin (MOX) removal. MOX is the most commonly used fluoroquinolones antibiotic, which is identified in wastewater and water due to its difficult dissociation by biological and wastewater treatment methods. The majority of fluoroquinolones antibiotics undergo incomplete metabolism within the body, resulting in the partial excretion of their pharmacologically active forms (> 50%). Additionally, a smaller proportion is excreted as phase I metabolites (formed through oxidation, reduction, or hydrolysis reactions) or phase II metabolites (formed through covalent conjugation with polar molecules such as glucuronic acid, sulfate, acetic acid, or amino acid)^17^. Fluoroquinolones are a class of synthetic antibacterial medicines that have been gaining increasing recognition and use. The use of quinolones, including second- and third-generation variants, is seeing a notable rise throughout several European nations. One example of a third-generation fluoroquinolone is moxifloxacin, as seen in Fig. 1. The use of third-generation quinolones in Europe in 2003 was widely acknowledged and accounted for 99.5% of their representation, as reported by Van Bambekeand Quinolones^18,19^. The primary method used in wastewater treatment facilities for the elimination of these persistent compounds is by sorption processes, rather than biodegradation^20^. In order to mitigate the development of resistance and minimize the occurrence of hazardous consequences, it is essential to use physico-chemical procedures for the removal of these antibiotics before their release into the environment, so we chose this type of antibiotic due to its critical importance.

**Figure 1:**
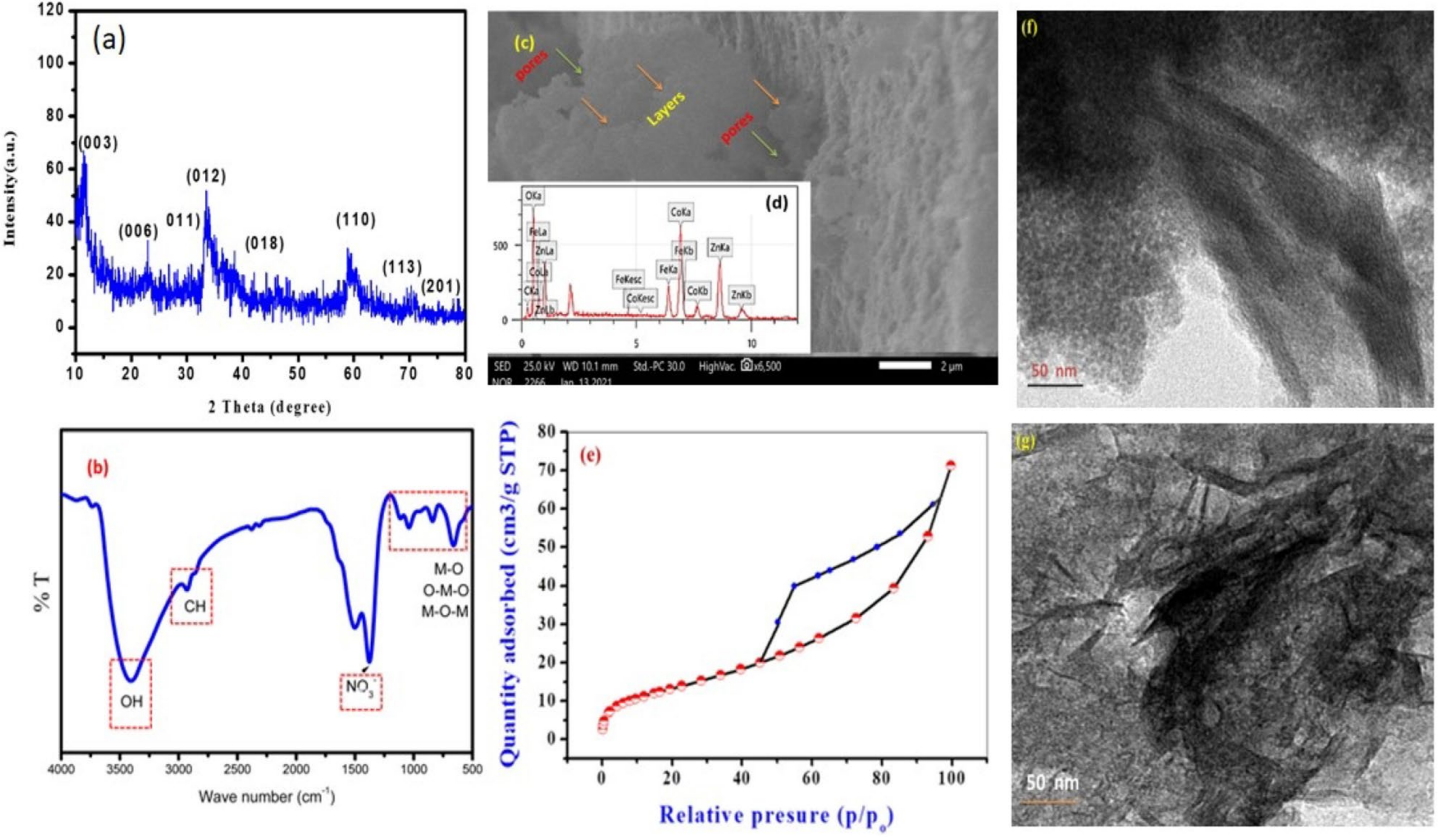
Characterization data of the prepared Zn–Co–Fe LDH (**a**) XRD, (**b**) FTIR, (**c**) SEM, (**d**) EDX, (**e**) Nitrogen adsorption desorption isotherm, (**f** and **g**) TEM images.

Besides water and wastewater applications, LDH can find wide applications in biomedical fields of research as a multifunctional materials due to not only their physico-chemical properties but also for their antimicrobial potential^8,21,22^. Most research in the open literature focuses on antibacterial properties on LDH material either as a single phase or in a composite with other metals^23,24^, such as silver and zero-valent iron, and/or metal oxides^25–29^, such as ZnO or CeO_2_. On the other hand, studying the impact of nanomaterials on fungus is a less explored field of research^30^. Investigating the antifungal properties of multifunctional LDH materials is an important field of research that can result in numerous economic and environmental gains^31,32^. For instance, the main factor causing significant economic loss while handling fruits after harvest is the growth of fungi^33^. Even when the most cutting-edge postharvest technology is used, some *Penicillium* strains can lead to severe fruit ailments after harvest, like grey and blue mold^33^. Apple and pear rot in storage is mostly caused by the *Penicillium* strain^34^. Additionally, *Penicillium* is thought to be the primary creator of the mycotoxin patulin, which is typically found in decaying food. Because fungi have evolved resistance to several common fungicides, including benzimidazoles and dicarboximides, it is challenging to control fungal development^35^. Even the previous important literature regarding the antimicrobial activity of LDH have reported valuable findings^8,15,36–41^ more investigations are required.

It is crucial to research innovative antifungal drugs that might take the place of present control methods in order to combat this resistance. In addition, fungi can cause series health problems for humans. For instance, *Mucor*mycosis is a severe but uncommon opportunistic fungal infection that spreads rapidly, necessitating prompt diagnosis and treatment to prevent high mortality and morbidity rates. *Mucor*omycota can as affect human through infection via trauma or injury^42^. Therefore, in the current work we extended our investigation to study the antifungal properties of the synthesized ZnCoFe LDH to explore its multifunctional potential. In this work were prepared this LDH using a simple co-precipitation methods. The prepared samples were characterized using XRD, FTIR, BET, TEM, SEM, and EDX. This LDH was investigated as a possible multifunctional adsorbent for wastewater applications to remove moxifloxacin (MOX). Moreover, for its antifungal activity against strains of *Penicillium notatum (P. notatum), Aspergillus flavus (A. flavus), Aspergillus fumigatus (A. fumigatus), Aspergillus niger (A. niger),* and *Mucor fungi* at various concentrations (1000–1.95 µg/mL) was investigated. This work paves the road towards further utilization of LDH materials for multiple applications especially in the fields of water disinfection, wastewater treatment, and biomedical applications. The work presented in this study is notable because to its capacity to synthesize low-cost LDH materials that can effectively remove fluoroquinolone waste from water. Additionally, these compounds exhibit antifungal activity, providing a dual mechanism for combating microbial contamination. Furthermore, this approach shows promise for addressing the issue of antimicrobial resistance.

## Materials and methods

### Materials

All chemical were used as received without any further purification. In addition, Bi-distilled water was employed throughout this work. Moxifloxacin (MOX) was purchased from (Hebe, China). Its full IUPAC name, CAS number and some important physical properties are summarized in Table 1. Zinc nitrate, cobalt nitrate and ferric nitrate were provided by SDFCL company, India. Sodium hydroxide and hydrochloric acid were purchased from Piochem and ACS BASIC Scharlau, respectively. Cyclohexamide, Amphotericin B, 0.8 M sorbitol, Oxoid Sabouraud's dextrose agar, Sabouraud's dextrose broth, Barium Sulfate 1.175%, and Sulfuric Acid 1% suspension were used throughout the antifungal study.

**Table 1:** Chemical and physical properties of MOX.

Common name	Moxifloxacin hydrochloride (MOX)	Reference
IUPAC name	7-[(4*aS*,7*aS*)-1,2,3,4,4*a*,5,7,7*a*-octahydropyrrolo[3,4-b]pyridin-6-yl]-1-cyclopropyl-6-fluoro-8-methoxy-4-oxoquinoline-3-carboxylic acid; hydrochloride	
CAS number	186826-86-8	
Therapeutic class		^43^
*Molecular formula*	C_21_H_25_ClFN_3_O_4_	
Density (g/cm^3^)	1.4 ± 0.1 g/cm^3^	
Solubility	Soluble in water (24 mg/ml), DMSO (88 mg/ml at 25 °C), and ethanol (< 1 mg/ml at 25 °C)	^43^
Molecular weight (g/mol)	437.9	
Molecular structure		^44^
		^44^

### Zn–Co–Fe LDH synthesis

In this study, Zn–Co–Fe LDH (2:2:1) was synthesized utilizing the co precipitation process. Zinc, cobalt, and ferric nitrate salts were dissolved in 50 mL of deionized water at a concentration of 0.4, 0.4 and 0.1 mol/L, respectively at room temperature. Sodium hydroxide (2 mol/L) was added drop wise under continuous stirring to the previously prepared salt solution until the pH of the solution reached 8.0. The resultant suspension was then aged for 24 h while being stirred to guarantee that the Zn–Co–Fe LDH completely precipitated at room temperature. The precipitate was filtered and rinsed with bi-distilled water until the pH of the filtrate reached 7.0. The final product, Zn–Co–Fe LDH, was then dried for 24 h at 50 °C in a vacuum oven drier^45^.

### Material characterization

The prepared LDH was analyzed by XRD (PANalytical Empyrean, Sweden) using Cu^2+^ Kα radiation (wave length 1.54060 Å) using a 40 kV accelerating voltage, a 30 mA current, a scan angle range of 10°–60°, scan step of 0.05° for identification and assessing the crystallinity of produced phases before and after the adsorption of MOX. FTIR spectroscopy was employed to ascertain the vibration of chemical bonds using a Bruker (vertex 70 FTIR/ FT-Raman, Germany) spectrophotometry (serial number 1341) having a frequency range of 400–4000 cm^−1^ and a potassium bromide disc. High-resolution transmission electron microscope (HRTEM, JEOL-JEM 2100, USA) and Field Emission Scanning Electron Microscope (FESEM) Germany (equipped with EDX, Quanta FEG250, Germany) were used to analyze the microstructure of the adsorbent and morphological characteristics. An automated surface analyzer (TriStar II 3020, Micrometrics, USA) to investigate the specific surface area and pore size distribution of the prepared adsorbent using nitrogen adsorption desorption.

### Adsorption study

During the adsorption study, the optimum conditions for adsorption were investigated by changing the solution's pH (3–11), initial concentration of MOX (5–100 mg L^−1^), Adsorbent dosage (0.01–0.05 g) and contact time (1–24 h). Filtration was used to remove the adsorbents from the solution following each experiment. The concentration of MOX was tracked by UV–visible spectrophotometer (UV-2600, Shimadz, Tokyo, Japan) at 290 nm^46,47^. To ensure their reproducibility, all tests were carried out in triplicate, and the average concentration was calculated applying SPSS version 16. *P* values under 0.05 were regarded as statistically significant values after computing the means and standard deviation (SD) values. The following Eq. (1) was used to calculate the effectiveness of MOX's adsorption on Zn–Co–Fe LDH^48^.

1 $$ Q = \frac{{\left( {C_{o} - C_{t} } \right)}}{{C_{o} }} \times 100 $$

where Q is the adsorptivity (%), C_o_ and C_t_ are the initial and final MOX concentration in (mg/L) at time t following adsorption (min). Equation (2) was applied to determine the MOX adsorption capacity at equilibrium qe (mg/g)^49^:

2 $$ q_{e} = \frac{{V\left( {C_{o} - C_{e} } \right)}}{W} $$

*C*_*o*_ stands for the initial concentration of pollutants ions before to adsorption in mg/L, and qe is the equilibrium adsorption capacity of the adsorbent in mg MOX/g LDH^50^. The equilibrium concentration of MOX pollutants is expressed in mg/L as Ce. V stands for the MOX solution volume in liters, and W represents the adsorbent weight in grams^51,52^.

### Fungal isolates and inoculum preparations

*Aspergillus flavus (A. flavus), Aspergillus fumigatus (A. fumigatus), Aspergillus niger (A. niger), and Penicillium notatum (P. notatum)* recent cultures were used to prepare the suspensions, which were then plated on Sabouraud's dextrose agar (SDA) and incubated at 35 °C for 24–48 h. 4–5 yeast colonies were transferred (using a sterile loop) into test tubes containing 5 mL of saline solution (0.9%) after incubation. Using a suspension of barium sulfates 1.175% and sulfuric acid 1%, the turbidity of the final inoculum was standardized (tube 0.5 on the McFarland scale, turbidity or standard tube). Approximately 1.5 × 10^8^ colony forming units per milliliter (CFU/mL) was the final concentration attained^53^.

### Determination of minimum inhibitory concentration (MIC)

The broth micro dilution method was used to determine the products' minimum inhibitory concentration (MIC) for the tested strains of *Mucor fungi (Mucormycosis or Black fungus), Aspergillus flavus (A. flavus), Aspergillus fumigatus (A. fumigatus), Aspergillus niger (A. niger), and Penicillium notatum (P. notatum).* A 96-well micro dilution plate with a "U" shaped bottom has 100 µl (100 µL) of Sabouraud's Dextrose broth medium (SDB) put into each well. The first horizontal row of the plate wells was then added with 100 µL of the tested nanomaterials emulsion. Concentrations of 1000–1.95 µg/mL were obtained using doubled serial dilutions, in which a 100 µL aliquot from the well with the highest concentration was transferred to the next well. Then, 10 µL of the inoculum suspension from the several tested strains was put to each well of the plate, where each column represented a distinct fungus strain. Positive control media (media containing the ingredients but without fungal strains) and negative control media were used in the presence of the common antifungal Cyclohexamide (media with the fungi but without nanomaterials). On SDA plates, each well held 100 µL of SDA with successive twofold dilutions of the tested LDH at various concentrations (1000, 500, 250, 125, 62.5, 31.25, 15.62, 7.81, 3.90, and 1.95 µg/mL). 10 µL solutions of 1.5 × 10^8^ fungal strain/mL were injected after the NPs had been diluted^53^. The plates were incubated for 24–48 h at 35 °C. Visual observation was used to determine if growth was there (or not) following the proper incubation period. It was thought that cell clusters or "buttons" would develop in the plate wells. The lowest concentration that generated clearly discernible fungal growth suppression was called the MIC.

### Minimum fungicidal concentration assay

We sub cultured 1 µL aliquots of the tested nanomaterials' MIC, MIC × 2, and MIC × 4 as well as amphotericin B and the fungal growth's negative control onto SDA-coated Petri plates in order to calculate the MFC. A reading was taken to evaluate the minimum fungicidal concentration (MFC) based on the growth of the controls after 24–48 h of incubation at 35 °C. The MFC was defined as the lowest product concentration that prevented the development of the various fungus species, producing fungicidal activity of either 50% or 99.9% as a consequence^54^. The findings of the triple biological activity experiments were represented as the arithmetic mean of the MIC and MFC. It was feasible to test the substance's potency using both dilution techniques, but it was impossible to tell if the chemical would actually kill the fungus or merely prevent it from growing. Depending on the fungus species being tested, small aliquots from each of the broth dilution tests are sub cultured on a rich solid medium and incubated for a predetermined period of time and temperature. The MFC is regarded as the lowest concentration of the material in which no discernible growth of bacteria has been seen. According to documentation created by the Chemical and Laboratory Standard Institute, the MFC is defined as the lowest concentration of the substance in which no appreciable bacterial growth has been seen^34^. Additionally, MFC could provide details on fungicide or fungiostatic activity. It is regarded as a compound fungicide if the MFC is equal to the MIC, but a fungiostatic if the MFC is higher than the MIC^55^.

### Sorbitol assay-effect of LDH on the cell wall of different tested fungal strains

To assess potential pathways involved in the antifungal activity of the test nanomaterials on the various fungus cell walls, the assay was carried out using medium with and without sorbitol (control). The culture medium (peptone water medium) received an addition of sorbitol at a concentration of (0.8 M sorbitol put on peptone water media 15 g/L). The test was carried out using a "U"-shaped 96-well plate using the micro-dilution technique. Readings were obtained on the fifth day of incubation after the plates had been aseptically sealed and incubated at 35 °C. The higher MIC values seen in the medium with added sorbitol compared to the regular medium implicated the cell wall as one of the sources of the higher MIC values since sorbitol can operate as an osmotic protection agent for fungal cell walls^56^.

### Agar diffusion method

Agar diffusion is a semi-quantitative experiment that involves adding a known amount of material containing LDH to an agar surface that has already been pre-inoculated with a certain quantity of fungi. Different methods can be used to apply the sample, such as disc diffusion, which involves impregnating discs made of sterile filter paper (6 mm) with the sample before applying them to the agar surface. Following inoculation, the samples diffuse into the agar medium, producing a gradient of concentration that is circular in shape. A growth inhibition zone will subsequently form all around the disc if the sample contains antifungal action after the fungus have multiplied. Some writers divide this millimeter-sized inhibitory zone into three categories: complete inhibition, moderate inhibition, and or no inhibition^57^.

### Antifungal assay

The antifungal activity of investigated nanomaterials was performed following the procedure reported by Agboola et al.^58^ against several randomly selected fungal isolates. The examined fungi were cultivated on SDA for 48 h at 35 °C before being suspended in physiological saline (0.9% NaCl) and adjusted to 1.5 × 10^8^ CFU. The tested nanomaterials were mixed with SDA at the tested concentration, and autoclaved at 121 °C for 15 min then maintained at 55 °C. The 1, 2, and 3% concentrations of LDH used in the test were prepared then the 20 mL of solidified Sabaroud agar medium was then added to sterilized petri plates^59,60^.

### Ethics approval and consent to participate

Not Applicable as the study not applied on human or animals study. The article does not include any studies on human participants or animals conducted by any of the authors.

## Results and discussion

### Material characterization

The characteristic reflections of LDH materials may be seen in the pattern of XRD for the Zn–Co–Fe LDH as shown in Fig. 1a. The Zn–Co–Fe LDH structure was demonstrated and validated by the presence of peaks at 11.3°, 23.09°, 33.6°, 35.97°, 43.44°, 59°, 63.68°, and 75.70, which correspond to (003), (006), (011), (012), (018), (110), (113), and (201), respectively, in accordance to reference cards (ICDD: 04-018-3495). The crystallite size of the prepared LDH was calculated using Deby–Sherrer's formula to be 3.52 nm. The FTIR spectrum of the prepared Zn–Co–Fe LDH (Fig. 1b) show a broad peak at 3400 cm^−1^, this peak can be referred to the OH^−^ stretching that caused by adsorbed H_2_O molecules^61^. Two peaks around 1510 cm^−1^ and 1368 cm^−1^ associated with the stretching mode of the NO_3_^–2^ anions^62^. The small peak at 2900 cm^−1^ may be originate from washing solvent (ethanol molecules)^63^.. Probably, ethanol molecules were trapped in the interlayer spacing between layers of the prepared LDH. The peaks below 1000 cm^−1^ could be attributed to the metal oxide (M–O, O–M–O and M–O–M) bond absorption where M is the metal (= Zn, Co or Fe)^64^. Scanning electron microscope images of the prepared LDH are shown in Fig. 1c. As shown, large layered structures can be observed with interparticle pores formed between the LDH aggregates. EDx analysis (Fig. 1d) reflects the purity of the prepared LDH with no signals other than Zn, Co and Fe signals. The surface area, mesoporosity and average pore size of the materials had been estimated by applying the nitrogen adsorption–desorption isotherms. The BET surface area of the prepared LDH was determined to be 155.90 m^2^/g, the mean pore diameter was 0.264 cm^2^/g and the pore volume was 8.416 nm. As shown in Fig. 1e the isotherm follows the type IV classification (according to IUPAC) with an H3 hysteresis loop^65^. This loop is common for layered materials with slit shaped pores, which is the case for LDH materials and such pores were observed before as shown in Fig. 1c). The TEM image (Fig. 1f) of the prepared LDH show the plate-like morphology of this LDH, which has been formed in a crumbled-like layered structure with large sheet length and width as illustrated in Fig. 1g. The layer size of the prepared LDH was approximately 140 nm.

### MOX adsorption investigation

Adsorption is one of most studied water and wastewater treatment method compared to other technologies such as photo-catalytic degradation and advanced oxidation processes^66,67^. This is due to its cheap cost, versatile nature for different pollutants and ease of operation^66,68^. Adsorption is an efficient technique that can handle different types of pollutants such as dyes, pharmaceuticals, herbicides, and/or heavy metals^69–72^. In this section, the results of MOX adsorption on the prepared LDH are illustrated and discussed.

### Effect of solution pH on MOX adsorption

**Figure 2:**
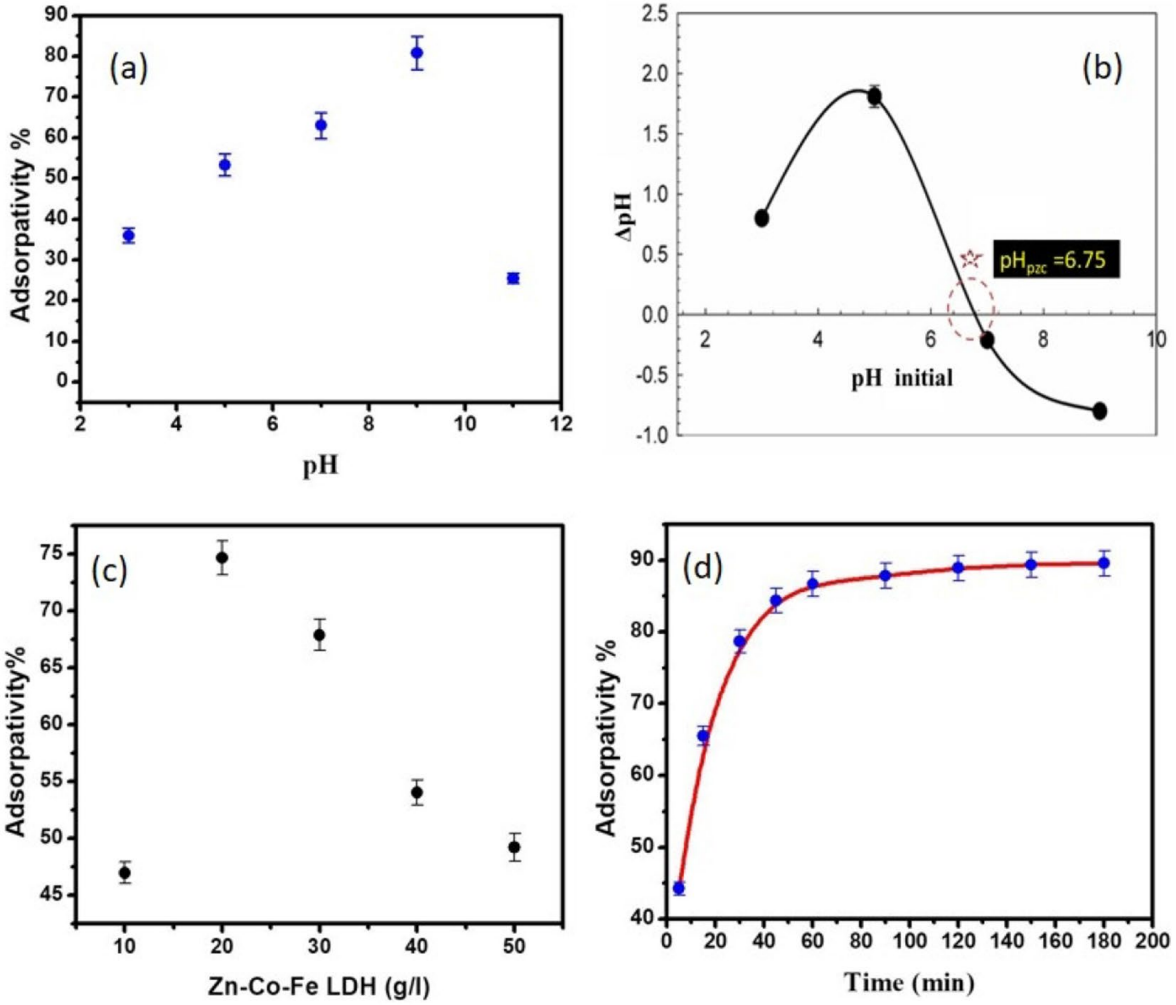
Investigation of main factors affecting MOX adsorption: (**a**) Adsorption% of MOX at various values of pH. (0.02 g adsorbent, 50 mL of 100 mg/L solution of MOX), (**b**) Point of zero charge curve, (**c**) effect of adsorption dose, (**d**) Effect of time on adsorptivity of MOX.

The chemistry of the antibiotic and the ionization state of the functional groups of the LDH are both influenced by the pH of the solution. That is why studying the effect of pH on adsorptivity (removal percent) is important. As shown in Fig. 2a, pH of the solution has a large effect on the ability of the synthesized material to remove MOX. As shown, the adsorptivity increased with increasing solution pH up to 80% at pH = 9. Further increase in solution pH led to a significant decrease in adsorptivity. To further understand this behavior, the point of zero charge of the LDH was determined as shown in Fig. 2b. As shown, before pH = 6.75, the LDH has a positive charge, while after this value the LDH has a negative charge. As for MOX, four pKa values were reported for this molecule which are pKa_1_ = 3.64, pKa_2_ = 5.05, pKa_3_ = 6.1–6.4, and pKa_4_ = 9.29–9.6^73^. In general, MOX molecules are positive at pH < 6.25 and act as zwitterion and negative ions in alkaline solutions^74^. Therefore, it can be assumed that irrespective of the negative charges on both the LDH and the MOX, at pH = 9 still maximum adsorption occurs. This reflects that the nature of adsorption doesn't rely on electrostatic attraction between different charges but rather other mechanisms may be causing the observed adsorption. More insights from adsorption isotherm study and kinetics are conducted to further assess the nature of adsorption between MOX and the prepared LDH.

### Effect of adsorption dose on MOX adsorption

The adsorptivity had been investigated by using different quantities of Zn–Co–Fe LDH dose per 20 ml MOX solution. As observed in Fig. 2c, the adsorption increased significantly with rising the dose of Zn–Co–Fe LDH up to 20 g/L. This can be attributed to the availability of active sites on the surface of the LDH, which lead to enhancement of MOX removal. The adsorption decrease with an increase in LDH dosage above 20 g/L. This is as a result of LDH particles aggregation, resulting in overall decreased removal efficiency^75^.

### Effect of contact time on MOX adsorption

The effect of contact time on the adsorption of MOX was investigated as shown in Fig. 2d. All experiments were conducted at an initial concentration of MOX = 100 mg/L, pH = 9 and LDH dose 20 g/L. As observed, the adsorptivity increases with contact time and reaches a plateau i.e. equilibrium in about 60 min. Further contact time doesn't result in any significant increase in adsorption, which can be attributed to the exhaustion of active sites on the LDH surface after reaching 60 min of contact time.

### Adsorption isotherm

**Figure 3:**
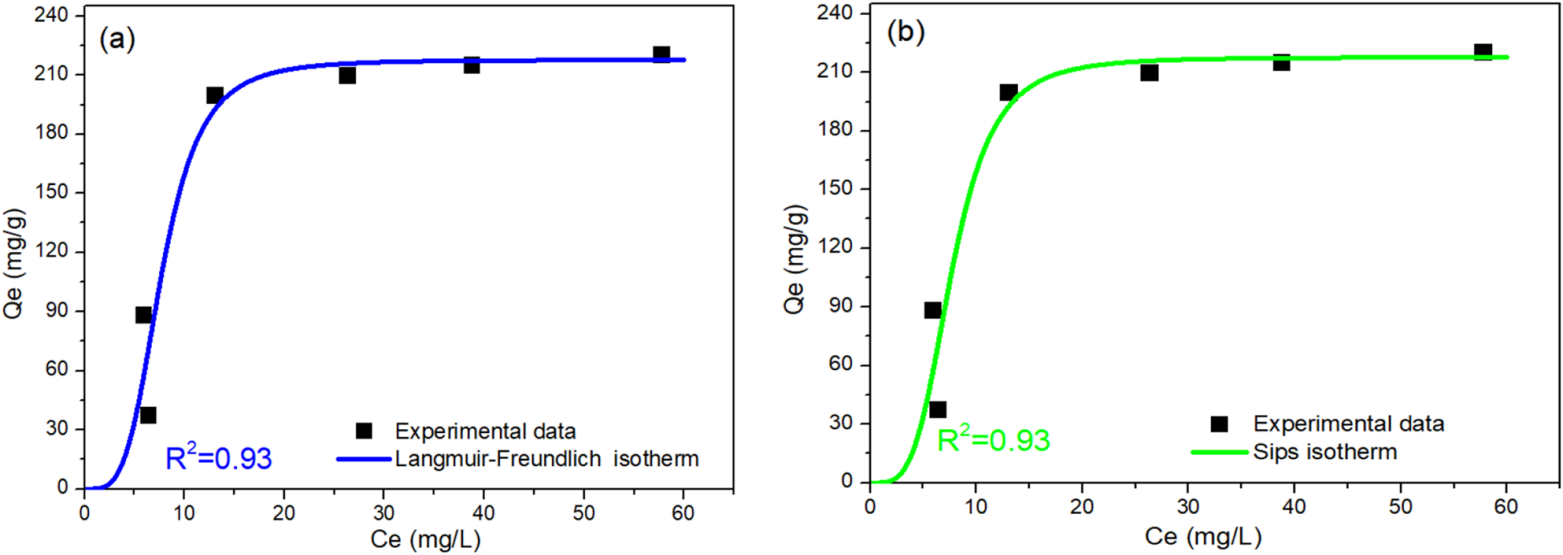
Adsorption isotherm data of MOX on the prepared LDH as fitted to (**a**) Langmuir–Freundlich and (**b**) Sips isotherm models.

In this study, numerous two-, three-, four-, and five-parameter isotherm models were investigated as shown in Table 2. Two-parameter models did not result in satisfactory fitting results as measured by the value of R^2^. In case of three-parameter models, Langmuir–Freundlich^79^ and Sips^14^ models showed a high R^2^ value of 0.93. Isotherm models of higher parameters did not result in higher R^2^ values. Even when the isotherm exponents of Fritz–Schlunder model were not restricted to conditions of being less than one, no higher values of R^2^ were obtained. Therefore, both Langmuir–Freundlich^79^ and Sips^14^ models were considered as the most satisfactory models to fit the experimental data, the results of which are shown in Fig. 3a,b, respectively. From Langmuir–Freundlich model, it can be estimated that the maximum adsorption capacity for Zn–Co–Fe LDH was 217.81 mg/g as shown in Table 2, which is similar to that obtained from Sips model (217.82). Langmuir–Freundlich model combines both Langmuir and Freundlich models and indicates that the LDH offers a heterogeneous surface for MOX adsorption^81^. Sips model is also a hybrid model that combines Langmuir and Freundlich models to predict the adsorption behavior of the heterogeneous structures^82^. More insights into the adsorption process can be gained from assessing the adsorption kinetics as discussed next.

**Table 2:** The non-linear isotherm models and the values of estimated parameters of MOX adsorption using Zn–Co–Fe LDH as adsorbent.

Isotherm models	Equation	Model parameters*		Values	*R*^2^
Two-parameters isotherm					
Langmuir^76^	$q_{e} = \frac{{q_{max} K_{L} C_{e} }}{{1 + K_{L} C_{e} }}$	*q*_*max*_ (mg/g)	Reflects monolayer formation	291.48	0.78
		*K*_L_ (L/mg)	Adsorption equilibrium constant	0.077	
Freundlich^77^	q_e_ = *K*_f_ C_e_^1/nf^	*K*_f_ (L/g)	Reflects the relative adsorption capacity	0.399	0.68
		1/*n*_f_ (−)	Constant for surface heterogeneity	49.13	
Temkin^78^	q_e_ = (R*T/b_*T*_*) Ln(A*_*T*_ * C_e_)R is the universal gas constant and T is the absolute temperature	b_T_	Temkin constant	34.56	0.76
		A_T_ (L/g)	Isotherm equilibrium binding constant	0.54	
Dubinin–Radushkevich^77^	q_e_ = (q_m_) exp (− K_ad_ ε^2^) ε = RT (1 + (1/C_e_))	*q*_*m*_ (mg/g)	Theoretical adsorption capacity	271.32	0.83
		K_ad_ (mol^2^/KJ^2^)	Related to adsorption energy	0.0016	
Three-parameters isotherm					
Langmuir–Freundlich^79^	$q_{e} = \frac{{q_{max} (K_{lf} C_{e} )^{{n_{s} }} }}{{1 + (K_{lf} C_{e} )^{{n_{s} }} }}$	*q*_*max*_ (mg/g)	max. adsorption capacity	217.81	0.93
		*K*_LF_ (L/mg)	Equilibrium constant	0.13	
		n_s_	Heterogeneity parameter	3.87	
Sips^14^	$q_{e} = \frac{{q_{max} K_{S} \left( {C_{e} } \right)^{{n_{s} }} }}{{1 + K_{S} (C_{e} )^{{n_{s} }} }}$	*q*_*max*_ (mg/g)	Sips constant for maximum adsorption capacity	217.82	0.93
		Ks (L/mg)	Equilibrium constant	0.00036	
		1/n_s_ (−)	Sips' exponent	3.88	
Redlich–Peterson^15^	${q}_{e}= \frac{{q}_{max} {C}_{e}}{1+ {{K}_{S} {(C}_{e})}^{{\beta }_{s}}}$	*q*_*max*_ (L/mg)	Isotherm constant	14.92	0.85
		K_s_ (mg/g)^g^	Isotherm constant	0.0028	
		β_s_ (−)	Isotherm constant	1.73	
Khan^79^	${q}_{e}= \frac{ {Q}_{m }{b}_{K } {C}_{e}}{[1+ {\left( {b}_{K } {C}_{e}\right)]}^{{a}_{k}}}$	${Q}_{m}$	Isotherm constant	5830.9	0.83
		${b}_{K}$	Isotherm constant	0.0029	
		${a}_{k}$	Isotherm constant	9.83	
Toth^14^	$q_{e} = \frac{{K_{e} C_{e} }}{{[1 + (K_{L} C_{e} )^{n} ]^{{1/n}} }}$	K_e_ (mol*L/mg*mg)	Tóth max. adsorption capacity	25.78	0.85
		K_L_ (L/mg)	Tóth equilibrium constant	0.0028	
		n (−)	Tóth model exponent	1.73	
Higher-parameters isotherm					
Baudu^79^	${q}_{e}= \frac{{q}_{max} {{b}_{o} ({C}_{e})}^{1+x+y}}{1+ {{b}_{o} ({C}_{e})}^{1+x}}$	*q*_*max*_ (mg/g)	Baudu max. adsorption capacity	5357.67	0.85
		*b*_o_(−)	equilibrium constant	0.0028	
		*X* (−)	Baudu parameter	− 0.73	
		*Y* (−)	Baudu parameter	0.73	
Fritz–Schlunder (IV)^80^	${q}_{e}= \frac{{q}_{mFSS} { {(C}_{e})}^{{m}_{1}}}{1+ {{K}_{1} {(C}_{e})}^{{m}_{2}}}$	${q}_{mFSS}$	Fritz–Schlunder maxium adsorption capacity (mg/g)	23.98	0.78
		${K}_{1}$	Model parameter	0.057	
		${m}_{1}$	Model exponent < 1	0.92	
		${m}_{2}$	Model exponent < 1	0.99	
Fritz–Schlunder (IV)(Unrestricted)^80^	${q}_{e}= \frac{{q}_{mFSS} { {(C}_{e})}^{{m}_{1}}}{1+ {{K}_{1} {(C}_{e})}^{{m}_{2}}}$	${q}_{mFSS}$	Fritz–Schlunder maxium adsorption capacity (mg/g)	0.1	0.93
		${K}_{1}$	Model parameter	0.00046	
		${m}_{1}$	Model exponent	3.72	
		${m}_{2}$	Model exponent	3.73	
Fritz–Schlunder (V)^79^	${q}_{e}= \frac{{q}_{mFSS} {K}_{1} { {(C}_{e})}^{{m}_{1}}}{1+ {{K}_{2} {(C}_{e})}^{{m}_{2}}}$	${q}_{mFSS}$	Fritz–Schlunder maximum adsorption capacity (mg/g)	11.22	0.78
		${K}_{1}$	Model parameter	2.14	
		${K}_{2}$	Model parameter	0.057	
		${m}_{1}$	Model exponent < 1	0.92	
		${m}_{2}$	Model exponent < 1	0.99	
Fritz–Schlunder (V) (Unrestricted)^79^	${q}_{e}= \frac{{q}_{mFSS} {K}_{1} { {(C}_{e})}^{{m}_{1}}}{1+ {{K}_{2} {(C}_{e})}^{{m}_{2}}}$	${q}_{mFSS}$	Fritz–Schlunder maxium adsorption capacity (mg/g)	0.74	0.93
		${K}_{1}$	Model parameter	0.14	
		${K}_{2}$	Model parameter	0.00045	
		${m}_{1}$	Model exponent	3.72	
		${m}_{2}$	Model exponent	3.74

### Adsorption kinetics

The adsorption equilibrium isotherm is crucial for defining how the adsorbate molecules spread between the solid and liquid phases when the adsorption process reaches equilibrium. The mechanism of adsorption based on the chemical and physical properties of the adsorbent, as well as the mass transfer procedure. Additionally, the adsorption process' kinetics study is important for designing adsorbents since it gives vital insights for understanding the mechanisms and the rate at which pollutants are adsorbed.

**Figure 4:**
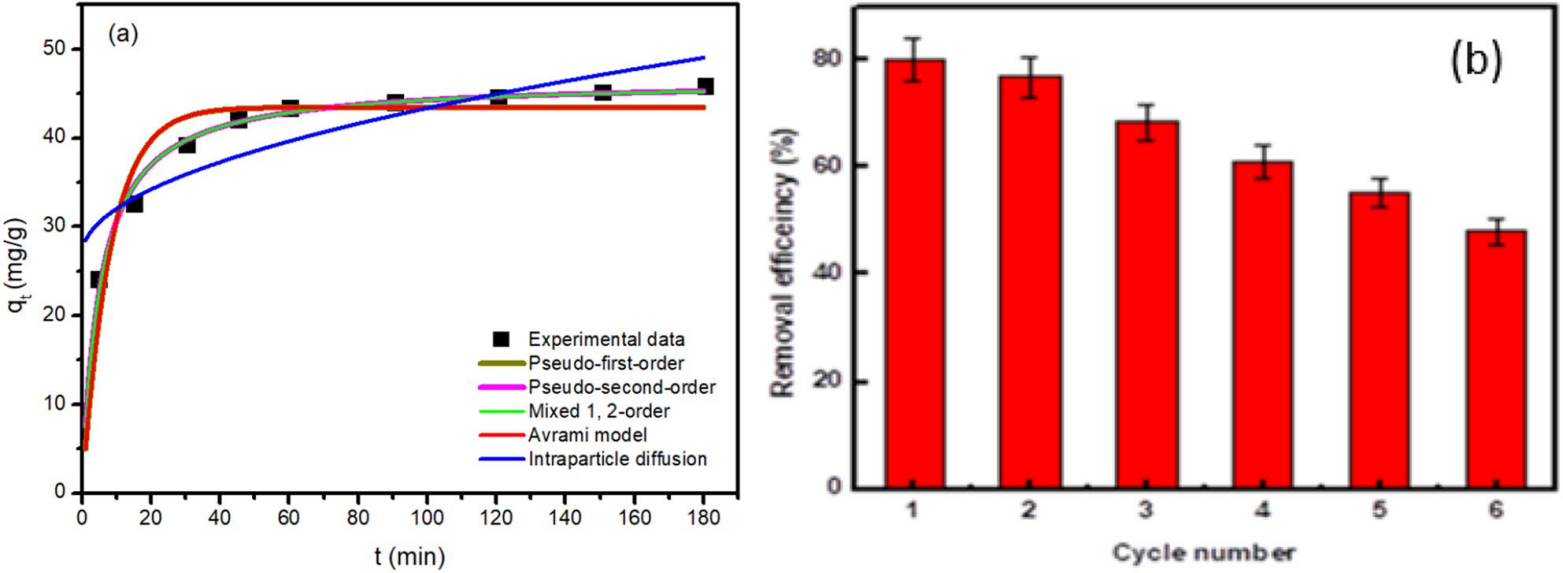
(**a**) kinetic data modelling for MOX adsorption on the Zn–Co–Fe LDH and (**b**) recyclability of Zn–Co–Fe LDH utilization for MOX adsorption.

In this study, five kinetic models (as shown in Table 3) were investigated to fit the experimental data of MOX adsorption. The fitting of these models to the data is shown in Fig. 4a. As illustrated in Table 3, the pseudo second order and mixed first and second order models results in the highest R^2^ values. The adequate fitting of the kinetic data to the pseudo second order model can indicate that the LDH has abundant active sites that can adsorb MOX molecules easily and quickly probably leading to a diffusion limited adsorption process^83,84^. The good fitting of the data with the mixed first and second order model further support the diffusion limited nature of the adsorption process^83^. Besides the adsorption kinetics and to reduce expenses, it is critical to determine the Zn–Co–Fe LDH performance LDH's during reuse. Zn–Co–Fe/LDH could adsorb 80% of MOX in the first cycle as shown in Fig. 4b. After five cycles, Zn–Co–Fe/elimination LDH's efficiency for MOX dropped to 55%.

**Table 3:** Kinetic study model fitting parameters.

Kinetic model	Kinetic equation	Model parameters	Values
Pseudo first order	${q}_{t}= {q}_{e} (1- {e}^{- {K}_{1}t}$) q_e_ is the adsorption capacity at equilibrium, k_1_ is the pseudo-first-order rate constant	K_1_ (min^−1^)	0.12
		q_e_ (mg/g)	43.48
		R^2^	0.86
Pseudo second order	${q}_{t}= \frac{{{q}_{e}}^{2} {K}_{2} t}{1+ {q}_{e} {K}_{2} t}$ k_2_ is the pseudo-second-order rate constant	K_2_ (g/(mg min))	0.004
		q_e_ (mg/g)	46.6
		R^2^	0.98
Mixed first and second order	${q}_{t}= {q}_{e}\frac{1- {e}^{- K t}}{1- {f}_{2} {e}^{- K t}}$ f_2_ is the mixed 1 st, 2nd order coefficient, k is the adsorption rate constant	K (min^−1^)	0.0022
		q_e_ (mg/g)	46.33
		f_2_ (g/(mg.min))	0.99
		R^2^	0.98
Avrami model	${q}_{t}= {q}_{e} \left(1-{\left[{e}^{- {K}_{av}t}\right]}^{{n}_{av}}\right)$ k_av_ is the Avrami rate constant and n_av_ is the model's component	K_av_ (min^−1^)	43.48
		n_av_ (−)	0.36
		q_e_ (mg/g)	0.34
		R^2^	0.86
Intraparticle diffusion	${q}_{t}= {K}_{ip} \sqrt{t}+ {C}_{ip}$ C_ip_ is intraparticle diffusion constant and k_ip_ is a measure of diffusion coefficient	k_ip_	1.66
		C_ip_	26.8
		R^2^	0.76

### Possible adsorption mechanisms

**Figure 5:**
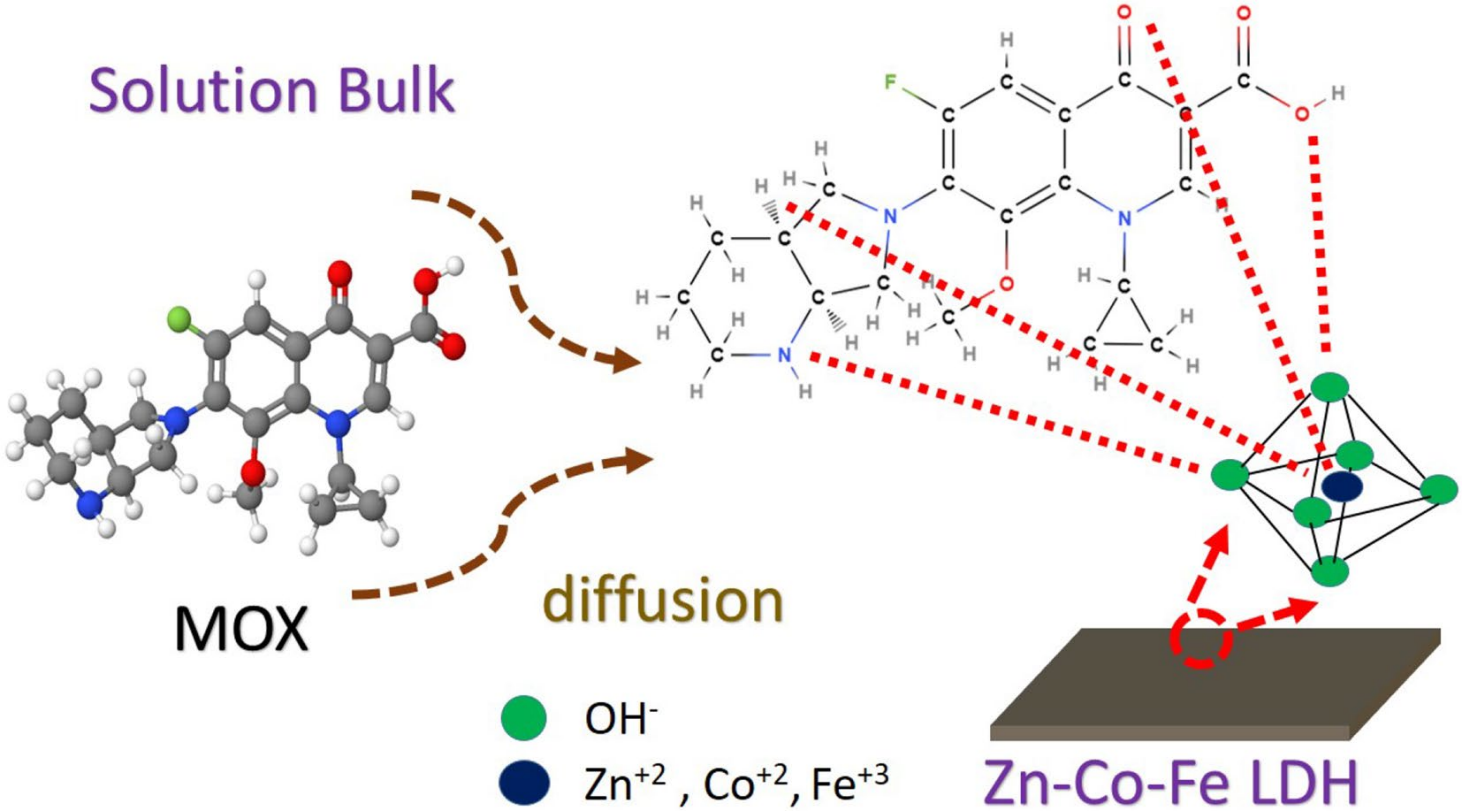
Possible adsorption mechanism(s) of MOX on Zn–Co–Fe LDH.

Zn–Co–Fe LDH is formed of a layered structure built from the octahedrons of Zn, Co, and Fe centers surrounded by OH^−^ groups as shown in Fig. 5. MOX molecules diffuse from the bulk of solution and interact with these octahedrons that form the large crumbled layers (as discussed in Fig. 1g). The interaction of MOX with such octahedrons is quick enough compared to the diffusion process an leads to a diffusion controlled adsorption as revealed by the kinetic study results. These octahedrons are not equivalent to each other and more interactions may occur in Zn or Co or Fe octahedrons leading to a heterogeneous layered surface as revealed by the isotherm study. This heterogeneous nature of the octahedrons can be attributed to the nature of the di-valent or tri-valent cation present in the center that can affect the strength if its oxygen bonds (oxygen atoms are present at the corners of the octahedron) and thereby affecting the overall performance of the octahedron as an active site for adsorption. When MOX molecules reach a specific octahedron site, several interaction mechanisms may occur. One possible mechanism is the interaction between positive cations (Zn, Co, and Fe) and MOX atoms having lone electron pairs. Another interaction mechanism is the hydrogen bonding possibly formed between hydrogen in MOX molecule and oxygen on the corners of the LDH octahedrons^85^. The rate of these interactions are considered to be fast enough compared to the rate of diffusion of MOX molecules thereby leading to a diffusion limited adsorption process as indicated by the PSO model. At equilibrium, it can be assumed that the interaction between MOX molecules and the metallic center at the octahedron is more dominant than that of the possible hydrogen bonding with oxygen atoms in the corners. This is because adsorption isotherm data revealed that the adsorption process occurs on a heterogonous surface probably due to the difference in chemical nature of the metallic center as opposed to hydrogen bonding that should have resulted in a homogenous surface because such bonding is a nonspecific bonding of nature that can occur to any oxygen atom in the corner of octahedrons.

At equilibrium, the synthesized LDH showed a maximum adsorption capacity of 217.81 mg/g while reaching equilibrium in 60 min. as shown in Table 4. This LDH is among the highest adsorbents that were reported for MOX adsorption in terms of adsorption capacity while being a simple and cheap adsorbent to prepare compared to carbon and metal–organic frameworks based adsorbents that are more expensive to prepare. LDH can cost 1–25 USD/kg while carbon based adsorbents and MOF can cost 0.5–132 USD/kg and 500–7300 USD/kg, respectively^86^. In addition, the prepared LDH has shown a relatively fast equilibrium time (60 min) compared to other adsorbents reported in the literature.

**Table 4:** Recent studies reporting the adsorption of MOX compared to the current study.

Adsorbent	BET surface area (m^2^/g)	pH	MOX Conc. (mg/L)	Equilibrium time (min)	Adsorbent mass (g/L)	Removal percent	Q_max_ (mg/g)	References
Biochar RH700	0.852	4	50	1440	10	40	1472	^74^
Biochar OW700	1.990	9	50	1440	10	20	1263	^74^
Zinc ferrite/activated carbon	723.5	6–7	–	5	0.01 g/20 mL	–	388.8	^87^
MOF-808-SIPA metal–organic framework	861.4	5	-	30	0.5	–	287.1	^88^
Agar coated magnetite nanoparticles	–	7	50	80	–	97	284.09	^89^
Zn–Co–Fe LDH	155.9	9	100	60	20	80	217.81	This work
Sulfonic acid-amide functionalized magnetic sodium alginate	15.95	7	–	120	–	–	149.57	^90^
Biochar RH300	0.144	9	50	240	10	96.18	61.01	^74^
Magnetite/pectin nanoparticles	–	7	5	30	–	89	28.57	^91^
Natural zeolite—clinoptilolite	–	–	0.2 mg/mL	–	0.2 g/20 mL	99	2.71	^92^
Mesoporous silica gel	435	–	–	–	–	–	0.2	^93^

### Antifungal study

Several papers in the open literature have considered the antibacterial properties of nanomaterials but studying the impact of nanomaterials on fungus is a less explored field of research^30^. This could be because bacterial systems are simpler than fungal ones^94^. However, fungal species can pose series threats to human health and need more research and attention to investigate the possible antifungal properties of nanomaterials. For instance, the infectious species of *Mucor* is responsible for immunosuppression that leads to *Mucor*mycosis, including delayed and severe neutropenia, serious hematological illness with or without stem cell transplantation, and the delayed use of corticosteroids^95^. Additional risk factors include iron overload, treatment with the iron chelator deferoxamine, poorly managed diabetes mellitus with or without diabetic ketoacidosis^96^. Additional risk factors include iron overload, treatment with the iron chelator deferoxamine, poorly managed diabetes mellitus with or without diabetic ketoacidosis^97^. Additionally, reports of intestinal *Mucor*mycosis have been made, particularly when spores are consumed^98^. The invasion of blood vessels and subsequent thrombosis that results in tissue necrosis are significant characteristics that the various kinds of *Mucor*mycosis have in common. Additionally, angio-invasion accounts for the widespread infection that is frequently seen in *Mucor*mycosis cases. It is noteworthy to note that *Mucor*mycosis differs from other forms of illnesses, such as conspicuous aspergillosis, in that it causes specific histological abnormalities, is rapidly evolving, and frequently results in widespread tissue rot. Furthermore, deferoxamine therapy, diabetic mellitus, and press overload are risk factors specifically for *Mucor*mycosis. It's crucial to look for new antifungal substances that can cure this fungus since it poses a threat^99^.

### Broth micro dilution method in two different media

**Figure 6:**
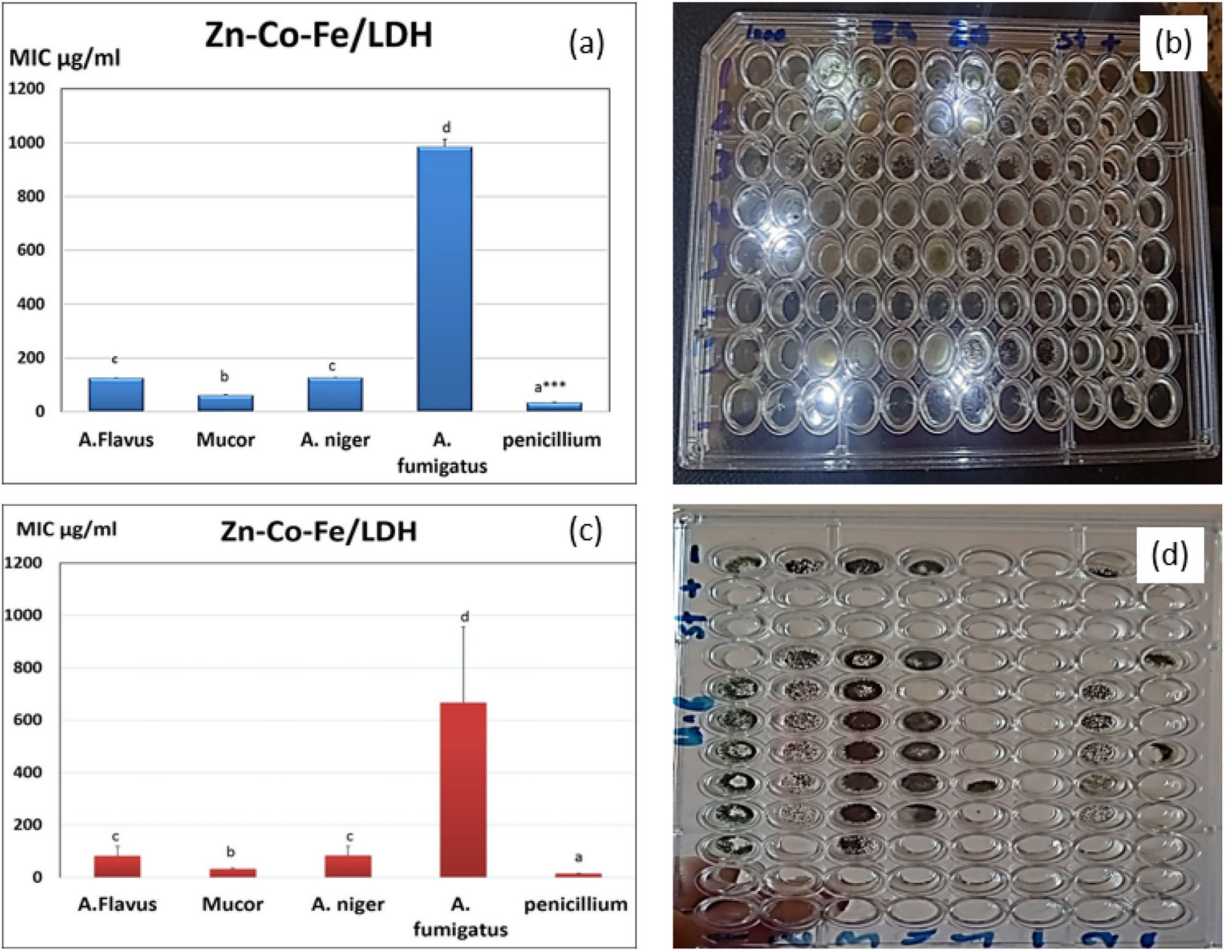
(**a**) MIC of Zn–Co–Fe LDH in SDB media against multiple fungal strains measured as mean ± SD, (**b**) photograph of the MIC results of Zn–Co–Fe LDH in SDB media against multiple fungal strains measured as mean ± SD, (**c**) MIC of Zn–Co–Fe LDH in Sorbitol/peptone water media against multiple fungal strains measured as mean ± SD, (**d**) photograph of MIC of Zn–Co–Fe LDH in Sorbitol/peptone water media against multiple fungal strains measured as mean ± SD.

The values of MIC of Zn–Co–Fe LDH is shown in Fig. 6. As shown Fig. 6a, shows the MIC values in SDB media (a photograph of the results in shown in Fig. 6b) while Fig. 6c shows the MIC of Zn–Co–Fe LDH in Sorbitol/peptone water media (a photograph of the results in shown in Fig. 6d). MIC was smaller and effective at against all fungal species especially against *Mucor* and *Penicillium* strains (33.3 and 61 µg/mL) respectively. LDH was showed also good antifungal activity against other species but in larger concentrations for MIC at (125 µg/mL) for *A. Niger and A. flavus* (980 µg/mL) against as shown in Fig. 6a,c.

For elucidation of the precise mechanism of action of the investigated compounds against various fungal strains, MIC measurements were repeated at various sorbitol medium. Lower effective MIC findings were suggestive of acting on the fungal cell wall. In comparison to the previously reported greater concentrations in a typical MIC, the findings of evaluating the MIC in various medium with more added sorbitol revealed that LDH became more active against *Penicillium* and *Mucor* with 16 and 32 µg/mL, respectively. An osmotic protector called sorbitol is utilized to keep fungus protoplasts stable. Unique fungal cell walls inhibitors all have the ability to reverse their antifungal effects in sorbitol-containing media^100^. In the presence of inhibitors of the fungal cell wall, sorbitol-protected cells can develop but in the absence of sorbitol, growth would be prevented. According to Frost et al.^100^, this impact may be seen as a decrease in the MIC value between the sorbitol-containing medium and the conventional medium^101^. Fungal cells can survive when osmotic destabilizing substances break the cell wall and cause cell wall rearrangements. The components that were examined appeared to impact the cell wall affecting its structure, blocking its synthesis, and causing cell death, while inhibiting spore germination, proliferation, and cellular respiration.^101,102^.

#### Minimum fungicidal concentrations (MFC)

**Figure 7:**
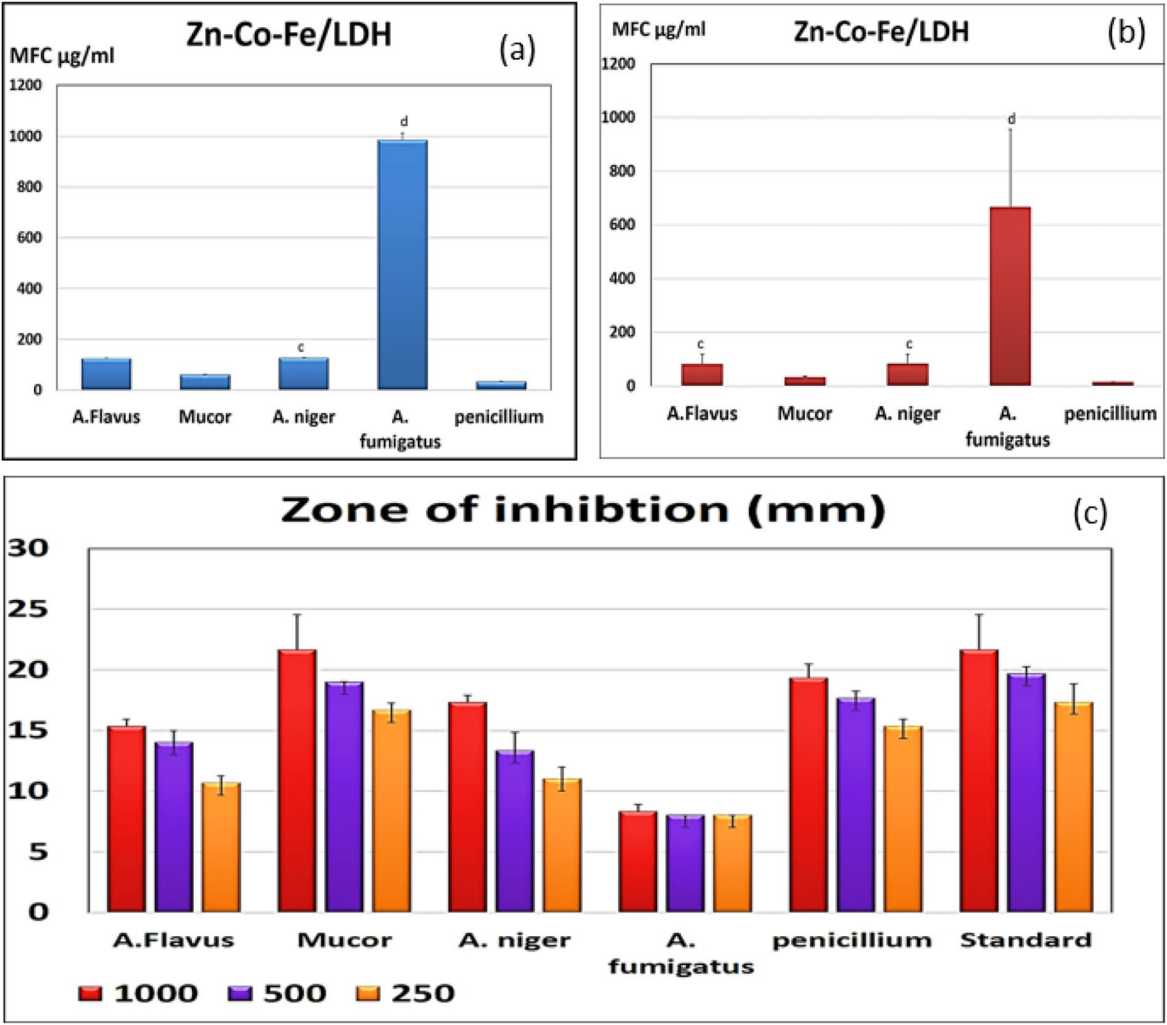
(**a**) MFC of Zn–Co–Fe LDH in SDA media against multiple fungal strains measured as mean ± SD, (**b**) MFC of Zn–Co–Fe LDH in Sorbitol/peptone water media against multiple fungal strains measured as mean ± SD, and (**c**) Zone of inhibition of Zn–Co–Fe LDH in SDA media against multiple fungal strains in three different concentrations of nanoparticles measured as mean ± SD.

The tested MFC results after using different media with added sorbitol concentrations showed also good effective fungicidal concentrations against *Mucor and* as showed in Fig. 7a,b. In all measure, the MICs were the same measures as the MFCs, demonstrating that the activity of the prepared Zn–Co–Fe LDH was fungicidal in *Penicillium* and *Mucor* species.

One of the most reliable techniques for determining the antifungal or antibacterial activity is the disc diffusion method. Zones of inhibition against several fungal strains were determined on SDA plates, and many concentrations are shown in Fig. 7c. When compared to the conventional antifungal for the rapidly growing fungus, LDH demonstrated a distinct zone of inhibition against *Penicillium* and *Mucor* when they were tested at doses (1000, 500, and 250 µg/ml) (chlorohexidine).

#### Antifungal activity (percent of inhibition %)

**Figure 8:**
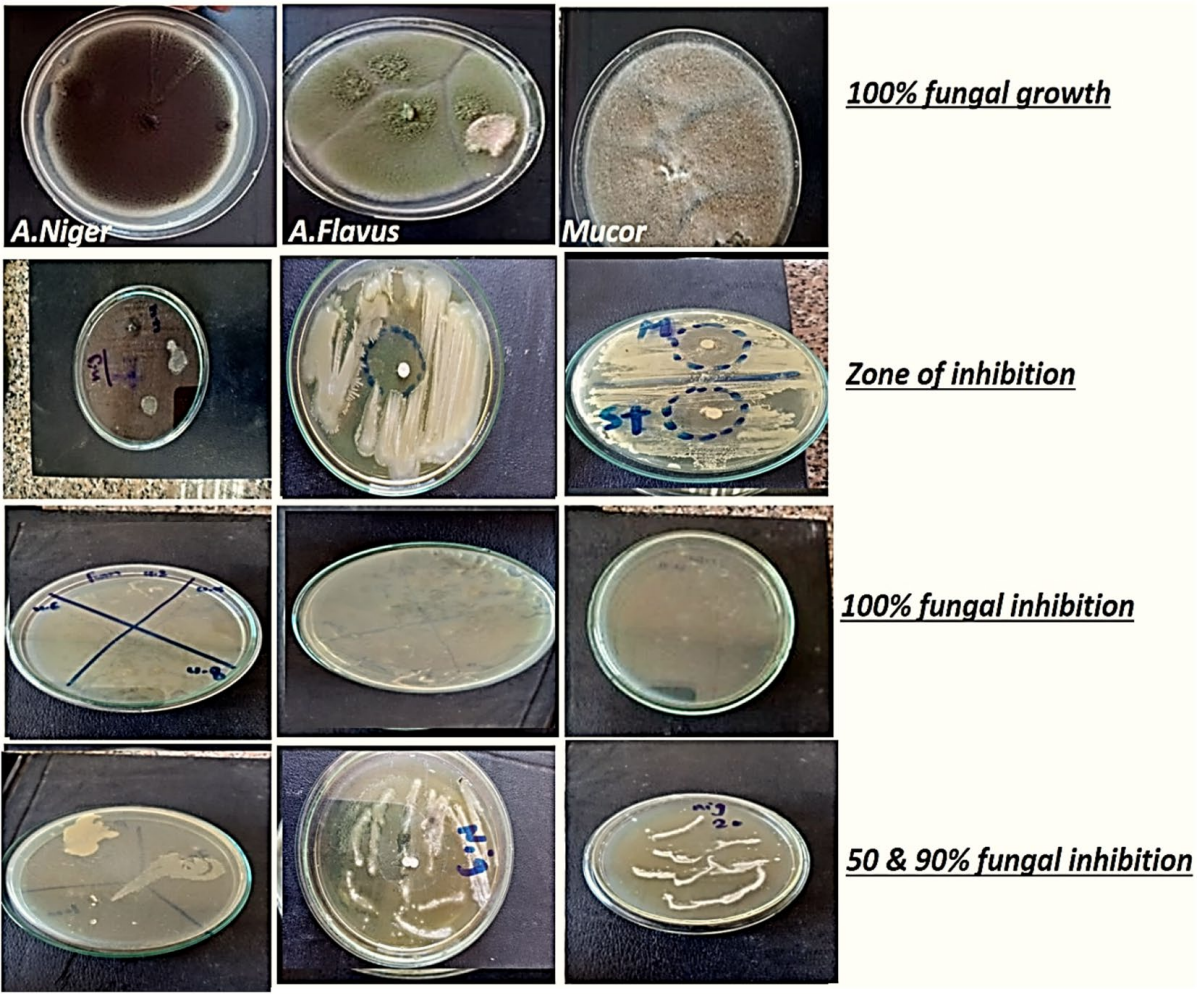
Photographic images showing antifungal activity percent % (representative samples for some tested fungal strains).

**Figure 9:**
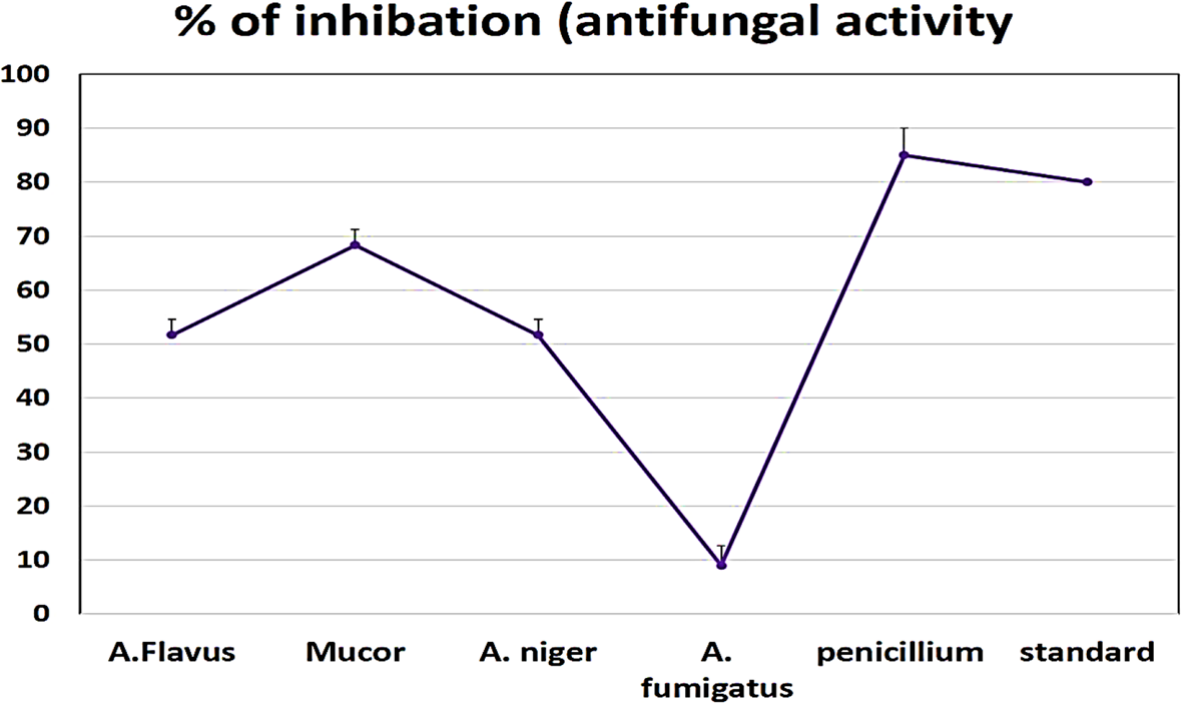
Zone of inhibition and antifungal activity percent % (representative samples for some tested fungal strains).

After adding the materials to the medium and estimating the antifungal activity, the percentage of fugal inhibition was calculated and shown in FigS. 8 and 9. With a 51.6% inhibition rate against *A. nigeria*, the tested LDH demonstrated modest antifungal efficacy. While against *Penicillium* and *Mucor* had demonstrated strong antifungal inhibition, it was attained (85 and 68.3%) even greater than the standard medication itself (80%). The samples' distinctive characteristics, including their wide surface area and uniform dispersion with prolonged or controlled release, account for their antifungal efficacy.

In these studies, as antifungal activity at good low concentration and against life -hreating fungal strains. The main antifungal activity in the synthesized LDH was due to the activity of the incorporated elements in-between the LDH layers (Zinc, Cobalt and Iron). According to preliminary studies, the formation of free radicals on the surface of synthesized nanomaterials' LDH and their subsequent damage to the lipids in bacterial or fungal cell membranes, which results in the leakage and breakdown of bacterial cell membrane, may be related to the antimicrobial activity of Zn nanomaterial^103,104^. To our knowledge, however, the impact and method of action of Zn nanoparticles on the development of fungi have not been well demonstrated.

The primary mechanism for the Zn–Co Fe LDH may depend on many mechanisms of action since the Zn element has shown a significant improvement in the antibacterial activity as a result of its distinctive qualities, such as its wide surface area. However,^105^ discovered that the toxicity of nano-sized and bulk ZnO against S. cerevisiae was equivalent. Uncertainty surrounds the mechanism behind the Zn nanoparticles' inhibitory effects on bacteria. According to several studies, integrating Zn nanomaterial into bacterial cells may cause the continual release of membrane proteins and lipids, which alters the permeability of the bacterial cells' membranes^105,106^, The application of zinc as a nanomaterial dramatically improved the nucleic acid and carbohydrate bands' intensity. These earlier findings point to or explain the primary antifungal action for the zinc integrated inside the LDH, which may impact cell functioning and, ultimately, because of the increase in the fungi's nucleic acid levels.

The increase in nucleic acid may be a result of the fungal hyphae's stress response. The self-protective mechanism against the zinc nanomaterial may be the cause of the increased glucose levels^107^. Others have also reported seeing more carbohydrates in fungus treated with zinc nanomaterial^108^. The excessive buildup of nucleic acid and carbohydrates may be the cause of the distorted structures in the hyphal cells. Compared to the previously described mechanisms for bacteria, our findings indicate a distinct mechanism for the inhibitory action of micro-zinc on fungus^109^.

The integration of cobalt into the LDH in this study also played a significant part in the antifungal activity. Co^+2^ and Co^+3^ coordination molecules' antiviral, antibacterial, anticancer, and antifungal properties have been well-documented^110^. Only Co^+3^ coordination molecules, nevertheless, continue to be of particular interest to researchers. Optically, isomer Co^+3^ complexes of the *N*,*N*-(di)donor ligand ethylenediamine [Co(en)_3_](NO_3_)_3_ with other metal ion complexes have demonstrated strong antibacterial activity in previous research.

Due to their therapeutic benefits as antiviral, antibacterial, anticancer, antifungal, antiparasitic, transferrin transport, and anti-inflammatory agents, cobalt complexes with organic ligands are particularly intriguing as possible candidates as antimicrobial agents. Significant antibacterial activity was shown by coordination compounds of Co^+3^ against resistant strains of *Pseudomonas, E. coli*, and reference strains of bacteria and fungus. Surfactant-Co^+3^ complexes, according to Kumar and Arunachalam^111^, shown strong antibacterial and antifungal action. Numerous fungi (*A. niger, N. crassa, and F. oxysporum*) were examined by Nagababu et al.^112^. They showed that some of these compounds had higher antibacterial activity than the tested antibiotics (gentamicin, streptomycin, penicillin, chloramphenicol, or ciprofloxacin). The studied complexes exhibited good antifungal activity^112^.

Co^+3^ in LDH shown potent antifungal action in the current investigation against several fungi strains and shared other components with other antifungal agents. In a prior investigation, cobalt showed that its effectiveness against *C. albicans* was the strongest. Due to the evaluated compounds' low MIC values and high antifungal inhibition percentage, our research implies that they have the potential to be used as antifungal agents.

We used a sorbitol experiment to evaluate the impact of the tested LDH on the fungal cell wall. The osmotic protection agent sorbitol stabilizes fungal protoplasts. In contrast to the lack of sorbitol, cells protected by this sugar alcohol can thrive in the presence of antimicrobial substances inhibiting the formation of fungal cell walls. The rise in MIC during the sorbitol studies may point to the cell wall as a potential site of the compound's action. The sample's sorbitol addition stresses the cell through osmotic pressure. Perhaps more of the substance then enters the cell. Similar MIC values for samples containing and excluding sorbitol show that the investigated substance has no impact on the production of the fungal cell wall. This antifungal action is dependent on the sterol molecules' ability to attach to the ergosterol-containing membrane of the fungal cell, which damages the cytoplasmic membrane. Following macromolecules, inert particles, bigger-diameter ions, and potassium ions leave the cell in that order. There is a metabolic imbalance and cell death^113^.

The presence of inhibition zone on culture media clearly indicates the biocidal activity of tested LDH mainly due to the incorporated elements as iron, zinc, and cobalt. The concentration of nanomaterials and the quantity of fungal spores both affect how much inhibition occurs. In a related study, iron oxide nanoparticles were shown to have promising antibacterial activity against a variety of human diseases. Green has been tested for its antifungal action against *Cladosporium cladosporoides and Aspergillus niger*, and the results showed that it is effective against both species even at low doses. Further, it was reported that *Aspergillus niger, Fusariumsolani, Escherichia coli, Bacillus subtilis, and Candida albicans* were all susceptible to the antifungal and antibacterial effects of iron oxide nanoparticles. Additionally, they claimed that iron oxide nanomaterial can function well as antibacterial, antifungal, or antimicrobial agents. The concentration of iron oxide that exhibits a greater zone of inhibition demonstrates the greatest activity index, according to data on iron oxide nanoparticle activity. The creation of numerous reactive oxygen species, such as superoxide (O_2_), hydroxyl radicals (^−^OH), singlet oxygen (O_2_), and hydrogen peroxide, results in oxidative stress (H_2_O_2_) Some metal oxide nanoparticles (NPs) release metal ions, which are then taken up by the cell membrane and interact with proteins and nucleic acids to cause damage to enzyme function. reported that reactive oxygen species generation, which results in oxidative stress and damages proteins and DNA in bacteria, is the source of iron oxide nanomaterial's antimicrobial action^114^ reported that oxidative stress is caused by iron nanosynthsis through the Fenton reaction and reactive oxygen species production. Since iron is a potent reductant, it causes functional groups in lipopolysaccharides and membrane proteins to break down. By oxidizing intracellular oxygen, iron nanomaterials can also induce oxidative damage via the Fenton reaction. These NPs pass across damaged membranes, further harming and killing cells^115–117^.

## Conclusions

Zn–Co–Fe LDH was investigated as a possible multifunctional adsorbent and was prepared using a simple co-precipitation method. This LDH was characterized using XRD, FTIR, SEM, TEM, EDX, and surface area analysis. The characterization data shows that this LDH possesses a typical layered structure and a surface area of 155.9 m^2^/g. For the MOX adsorption study, the maximum removal efficiency was calculated to be 217.81 mg/g and the equilibrium time was measured to be 60 min. The interaction between MOX molecules and this LDH can be based on the possible bond formation between the metallic center of the octahedron and the atoms with a lone pair of electrons in the MOX structure leading to a heterogeneous surface of the LDH. As for the antifungal investigation, Zn–Co–Fe LDH showed excellent fungicidal activity against different pathogenic fungal strains, especially the most dangerous *Mucor* fungi that’s responsible for *Mucor*mycosis, which is a fatal serious infection that causes mortality in people. *Fungicidal activity (MIC and MFC) for the LDH against Mucor* and *Penicillium* strains was 33.3 and 61 µg/mL, 125 µg/mL for *A. Niger and A. Flavus* (980 µg/mL). MIC in sorbitol became more active against *Penicillium* and *Mucor* with 16 and 32 µg/mL. *Penicillium* and *Mucor* had demonstrated strong antifungal inhibition (85% and 68.3%). These results show that the ZnCoFe LDH can be considered a possible multifunctional adsorbent with a relatively high adsorption capacity for MOX in a reasonable equilibrium time. In addition, it can be considered a promising antifungal agent against numerous pathogens, which reflects its multi-functionality as a 2D nanomaterial. These results pave the road for further investigation of this and other LDH samples with possibly different morphologies and chemistries as promising multifunctional adsorbents, antimicrobial agents, and disinfectants, while also being cheap and efficient candidates.

## Data Availability

The datasets used and/or analyzed during the current study available from the corresponding author on reasonable request.

## Abbreviations

LDH: Layered double hydroxides
MOX: Moxifloxacin
XRD: X-Ray diffraction
FTIR: Fourier-transform infrared spectroscopy
BET: Brunauer–Emmett–Teller
TEM: Transmission electron microscopy
HRTEM: High-resolution transmission electron microscopy
SEM: Scanning electron microscopy
FESEM: Field emission scanning electron microscopy
EDX: Energy-dispersive X-ray spectroscopy
DMSO: Dimethylsulfoxide
SD: Standard deviation
SDA: Sabouraud's dextrose agar
CFU: Colony forming units
MIC: Minimum inhibitory concentration
SDB: Sabouraud's dextrose broth medium
MFC: Minimum fungicidal concentration
ICDD: International Centre for Diffraction Data
USD: United States dollar
MOF: Metal organic frameworks
